# Cross-Species Evidence for Psilocin-Induced Visual Distortions: Apparent Motion Is Perceived by Both Humans and Rats

**DOI:** 10.1016/j.bpsgos.2025.100524

**Published:** 2025-05-02

**Authors:** Čestmír Vejmola, Klára Šíchová, Kateřina Syrová, Lucie Janečková, Vlastimil Koudelka, Michael Tesař, Marek Nikolič, Michaela Viktorinová, Filip Tylš, Jakub Korčák, Vojtěch Viktorin, Eduard Kelemen, Tereza Nekovářová, Martin Brunovský, Jiří Horáček, Martin Kuchař, Tomáš Páleníček

**Affiliations:** a Psychedelic Research Center, National Institute of Mental Health, Klecany, Czechia; b Third Faculty of Medicine, Charles University, Prague, Czechia; c Forensic Laboratory of Biologically Active Substances, Department of Chemistry of Natural Compounds, University of Chemistry and Technology, Prague, Czechia; d Clinical Research Program, National Institute of Mental Health, Klecany, Czechia; e Faculty of Science, Charles University, Prague, Czechia; f Sleep and Chronobiology Research Center, National Institute of Mental Health, Klecany, Czechia; g Center for Advanced Studies of Brain and Consciousness, National Institute of Mental Health, Klecany, Czechia

**Keywords:** Human, Psilocin, Psychedelics, Rat, Vision, Visual hallucinations

## Abstract

**Background:**

Psychedelics, particularly psilocin, are increasingly being studied for their mind-altering effects and potential therapeutic applications in psychiatry. Visual hallucinations, especially the illusion of motion in static images, are a hallmark of their action. Despite growing interest, the underlying mechanisms remain poorly understood, as their systematic evaluation in both humans and animals is challenging.

**Methods:**

To investigate psilocin-induced visual distortions, we designed a 2-choice visual discrimination task. Human participants and male rats indicated whether an image appeared static or moving while the image either actually moved or did not. In humans, performance was compared with self-reported hallucination intensity, Altered States of Consciousness scale scores, and psilocin plasma levels. Rats were tested in 2 distinct tasks, a luminance-based task and a motion-based task. Their performance was evaluated alongside decision time.

**Results:**

Both species exhibited significant impairment in distinguishing static from dynamic visual stimuli while under psilocin’s influence. In humans, this impairment followed the time course of psilocin plasma levels and hallucination intensity. In rats, psilocin selectively impaired performance in the motion-based task, while performance in the luminance-based task remained intact, indicating a specific effect on visual perception. Decision time was linked to discrimination impairment.

**Conclusions:**

Psilocin impaired static-dynamic discrimination in both species, providing the first evidence that rats experience visual distortions similar to those reported by humans. The correlations between discrimination impairment, psilocin levels, and hallucination intensity in humans reinforce psilocin’s effects on visual perception. This approach provides a valuable tool for investigating the neurobiology of altered visual perception in drug-induced states and psychiatric conditions.

## Plain Language Summary

In this study, we explored how psilocin, a compound derived from psilocybin in magic mushrooms, alters visual perception in humans and rats. Using a visual discrimination task, both species were tested on their ability to distinguish static from dynamic images. Psilocin caused humans to misclassify static images as dynamic and induced similar visual distortions in rats. This is the first study to demonstrate that rats experience psilocin-induced visual distortions comparable to those reported by humans, thereby providing a valuable foundation for further research on visual alterations across species.

Visual hallucinations range from distorted perceptions of real objects to the appearance of nonexistent stimuli. Psilocybin, the active compound in magic mushrooms (1), is metabolized in the digestive tract into psilocin (2), which interacts with the serotonergic system, primarily through 5-HT_2A_ receptor agonism, to produce its psychoactive effects (3,4). These receptors are abundant in the visual pathway (5, 6, 7, 8, 9), and recent molecular, pharmacological, and neuroimaging studies have highlighted their role in visual processing and visual hallucinations (10,11). Their alterations have also been associated with visual hallucinations in Parkinson’s disease (11,12) and schizophrenia (13). Conversely, blocking 5-HT_2A_ receptors has been shown to reduce hallucinations in these conditions (14,15).

Psychedelics such as lysergic acid diethylamide (LSD), psilocin, mescaline, and DMT frequently induce motion-like distortions in stationary objects, often described as breathing, warping, undulating, or swirling (16, 17, 18, 19, 20, 21, 22). At moderate doses, these effects begin as rhythmic contour oscillations and progress to vibrations, geometric transformations, and spatial distortions (23). At higher doses, reality becomes increasingly unstable, with complex, vivid 3-dimensional imagery, including fractal-like structures and immersive landscapes. Individuals may report experiencing tunnels, spirals, or shifting grids, culminating in fully immersive visionary states, often with introspective or mystical themes (16, 17, 18, 19, 20, 21, 22). These effects are commonly assessed using the Altered States of Consciousness (ASC) scale (24,25) and the Hallucinogen Rating Scale (26), which show that hallucinatory intensity correlates with psilocybin dose, reaching up to 60% of the maximum theoretical scale for perceptual alterations at high doses (27,28).

Animals exposed to psychedelics display disorganized behavior, suggesting hallucinatory-like experiences. Psychedelics disrupt spiders’ web spinning (29,30), fish swimming patterns (31, 32, 33, 34, 35, 36), and mice’s head-twitch response (37, 38, 39, 40) or performance on sensorimotor tests (36)—effects that could be explained by sensory deficits (41). Psychedelic-treated cats visually tracked, reached for, or recoiled from nonexistent objects—behaviors strongly indicative of visual hallucinations (42, 43, 44, 45). Similarly, dogs bark at or chase unseen stimuli (46,47), pigs display disoriented movements (48), and primates have been observed grasping at imaginary objects (49,50), suggesting that psychedelics interfere with the perception of reality across species. Despite these observations, no study has yet attempted to systematically and objectively measure visual hallucinations in animals.

While rats have traditionally been considered poor models for vision research, recent studies have shown that their visual processing is highly advanced (51, 52, 53, 54). The rodent visual system is organized into 2 clusters of reciprocally connected areas, which closely resemble the primate ventral and dorsal streams in both anatomy (55,56) and function (57,58). Motion perception in rats is processed hierarchically, first in primary visual cortex, and is then integrated into higher extrastriate areas, paralleling primate vision (59, 60, 61). These findings suggest that rats are suitable subjects for studying visual perception (62).

To assess psilocin-induced visual distortions, we developed a 2-choice visual discrimination task in both species, hypothesizing that psilocin would impair the ability to distinguish static from dynamic images, leading to a higher tendency to misclassify static stimuli as dynamic. In humans, performance was analyzed together with self-reported hallucination intensity, while in rats, decision time served as an objective behavioral marker. To exclude other cognitive effects of psilocin (63,64), we utilized a visual discrimination maze that requires active problem solving in rats (65, 66, 67, 68) and incorporated 2 distinct tasks to assess different aspects of visual involvement. This is the first study to use an objective task to quantify drug-induced visual distortions in animals, thereby providing a cross-species comparison of hallucinatory effects.

## Methods and Materials

### Human Experiment

#### Study Design and Participants

This study was part of a double-blind, placebo-controlled, crossover clinical trial (EudraCT No. 2012-004579-37), approved by the Ethical Committee of the National Institute of Mental Health and the State Institute for Drug Control. Twenty-one healthy volunteers (10 women), ages 28 to 53 years (mean = 37 ± 6.1), provided written informed consent. Psilocybin was administered orally at 0.26 mg/kg [equivalent to a full-hallucinogenic high dose (27,28)] using 1 mg and 5 mg capsules prepared at the Institute of Clinical and Experimental Medicine, Prague. Identical placebo capsules contained tritici amylum. The minimum washout period between sessions was 28 days (mean = 49 days). For more details on dose preparation, study design, and administration, see the Supplement and Bravermanová *et al.* (69).

#### Visual Task and Stimuli

**Figure 1:**
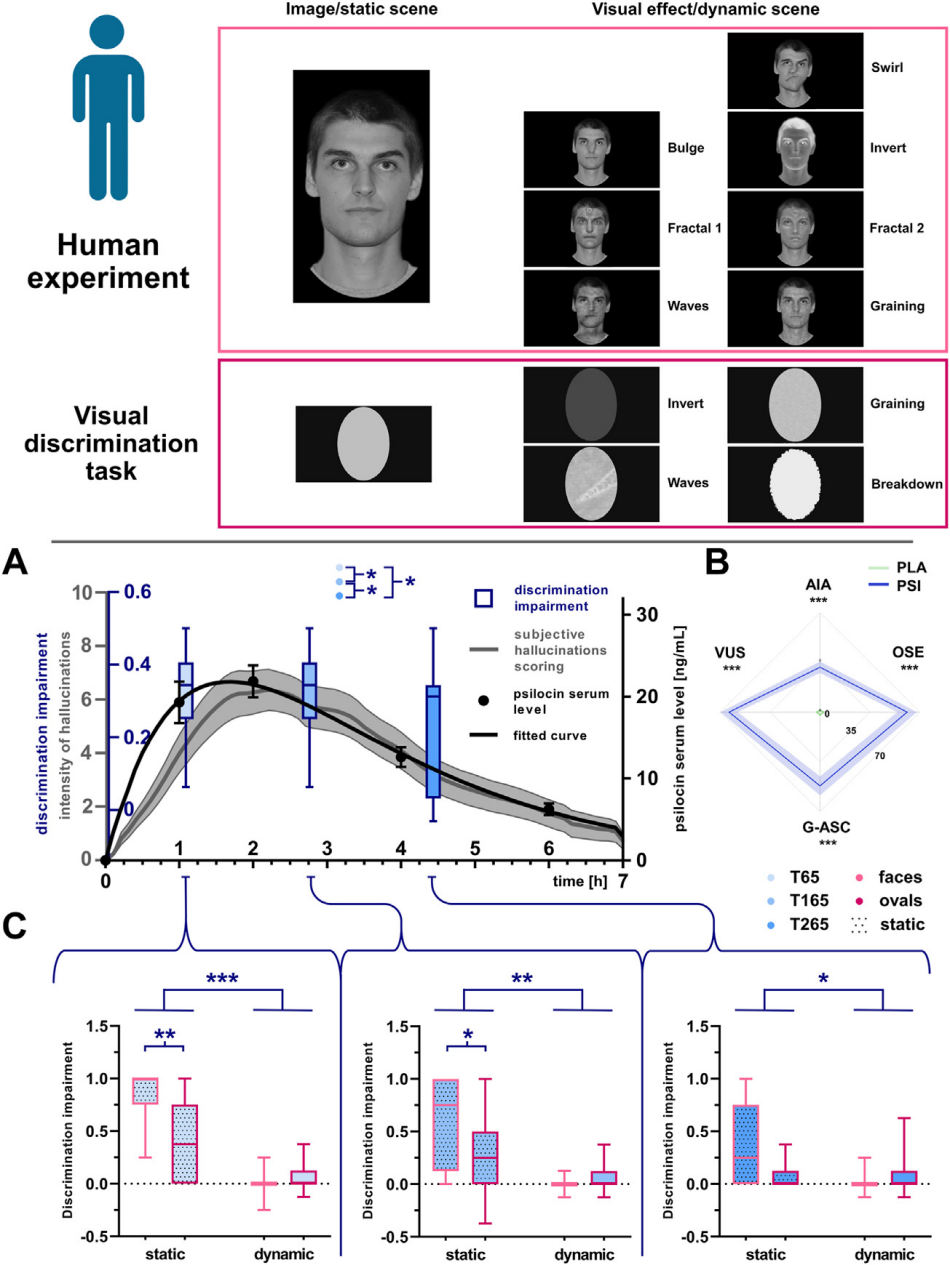
Human experiment. Top: Visual cues consisted of static (left column) and dynamic (right column) stimuli. Faces are outlined in pink, and ellipsoids are outlined in purple. Dynamic cues are shown at their maximum visual effect. To preserve anonymity, the effects are shown only on the face of the main author. **(A)** Effects of psilocin in humans. Boxplots (blue) represent discrimination impairment at 3 time points: 65 minutes (light blue), 165 minutes (mid blue), and 265 minutes (blue) after administration, pooled for faces and ellipsoids (median line, whiskers minimum to maximum). The gray line shows self-reported hallucination intensity (mean ± SEM), while black dots represent psilocin serum levels (mean ± SEM). The black curve follows a first-order absorption model. **(B)** The radar chart illustrates ASC scale scores, with the green line representing placebo and the blue line representing psilocybin. **(C)** Discrimination impairment split by cue type: static (dotted fill) and dynamic (solid fill) for faces (pink bordered) and ellipsoids (purple bordered). Statistical significance: ∗*p* < .05, ∗∗*p* < .01, ∗∗∗*p* < .001. AIA, dread of ego dissolution; ASC, Altered States of Consciousness; G-ASC, overall magnitude encompassing all questionnaire items; OSE, oceanic boundlessness; PLA, placebo; PSI, psilocybin; VUS, visionary restructuralization.

Participants performed a visual discrimination task at 65, 165, and 265 minutes post-psilocybin administration. The task, which lasted 6 minutes per session, involved presentation of either static or dynamic visual scenes for 8 seconds. Dynamic scenes were created using Resolume Arena 6 software, applying visual effects to either grayscale human faces or a white ellipsoid on a black background (Figure 1, top). Stimuli were presented randomly, each appearing 8 times. Participants responded by selecting whether the image was “moving” or “not moving” using a mouse. The experiment was conducted using OpenSesame software on a 22-inch LCD screen at a 70 cm viewing distance. See the Supplement for details on visual effects and trial randomization.

#### Acute Subjective Drug Effects

Self-reported effects were measured at 380 minutes postingestion using the ASC scale (25), which includes 72 items assessing oceanic boundlessness (OSE), dread of ego dissolution (AIA), visionary restructuralization (VUS), and general ASC (G-ASC) (Figure 1B). Participants also plotted hallucination intensity over time using a continuous visual analog scale (0–7 hours, 0–10 intensity). Graph digitization was performed using WebPlotDigitizer (70,71). See the Supplement for details on data digitization and analysis.

#### Psilocin Quantification

Blood samples were collected at 1, 2, 4, 6, and 24 hours postingestion, processed, and stored at −80 °C. Free and total psilocin concentrations were analyzed using liquid chromatography–tandem mass spectrometry. The full analytical protocol, including sample preparation, chromatography conditions, and pharmacokinetic modeling, is available in the Supplement.

### Rat Experiment

#### Animals

Ten male Long-Evans rats were housed in pairs in enriched plastic cages with chew toys and polyvinyl chloride (PVC) tubes, maintained at 22 °C, 40% humidity, under a 12-hour light/dark cycle. Food restriction ensured motivation for reward-based learning, with weights maintained above 90% of baseline. Water was available ad libitum. The study was approved by the Czech National Committee for the Care and Use of Laboratory Animals (MEYSCR36607/2018–4).

#### Drugs

Psilocin (0.3 mg/kg) or saline was subcutaneously injected in a 2 mL/kg volume 30 minutes before testing. Psilocin was synthesized and supplied by the Forensic Laboratory of Biologically Active Compounds, University of Chemistry and Technology, Prague and freshly prepared in 0.9% NaCl with glacial acetic acid (5 μL/20 mL). Dose selection was based on preliminary tests evaluating behavioral engagement across increasing doses (see the Supplement).

#### Visual Task and Training

**Figure 2:**
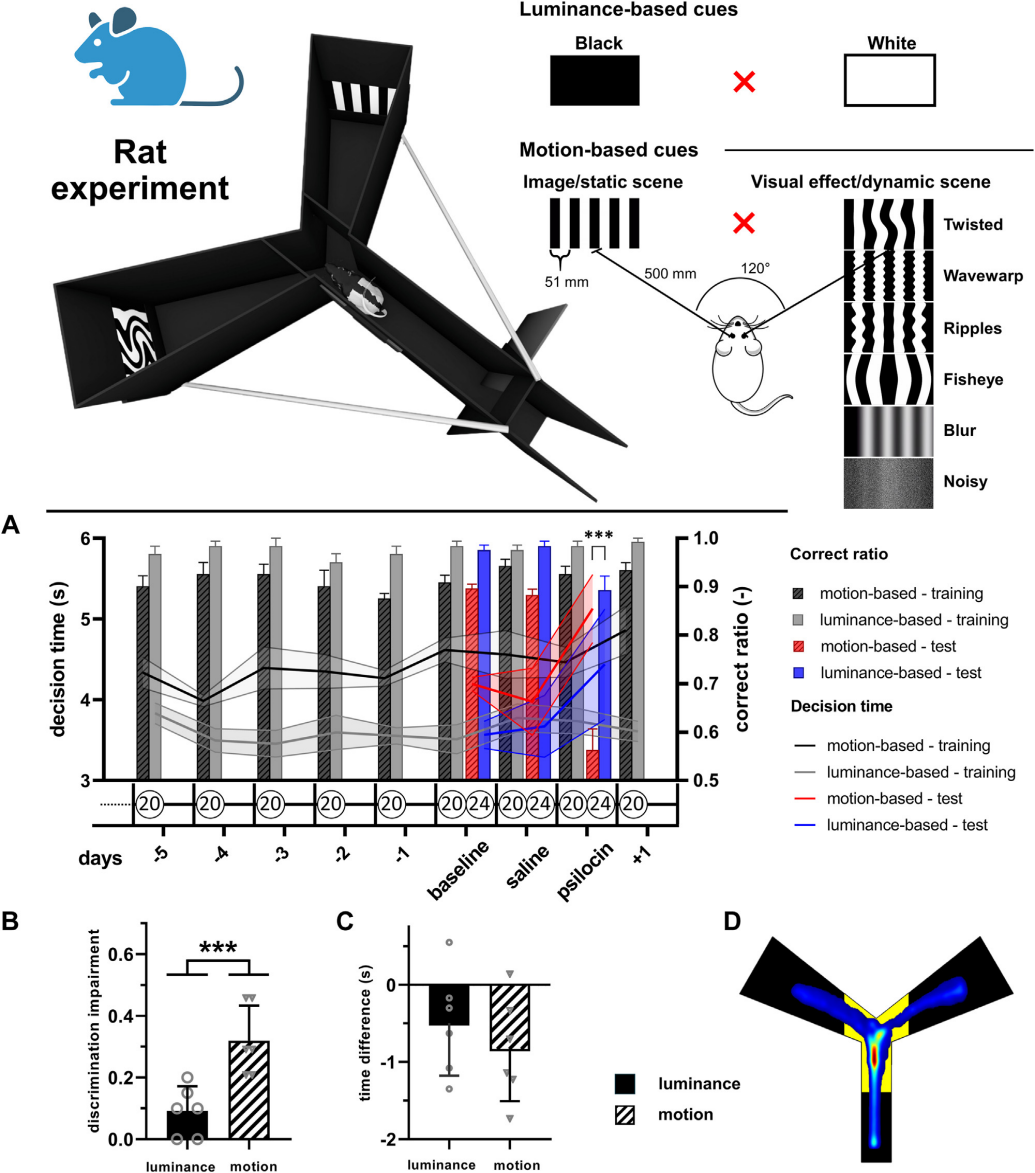
Rat experiment. Top: A 3-dimensional schematic of the polyvinyl chloride–manufactured maze and visual cues used in luminance-based and motion-based tasks. The maze consists of a 62-cm entrance corridor with a sliding door leading to 2 arms positioned at a 120° angle. Each arm is 50 cm long, initially 10 cm wide and expanding to 23 cm at the end, where visual stimuli are presented on screens positioned 3 cm above the floor. Rewards were delivered via aluminum tubes ending 1 cm above the floor. Dynamic cues were generated by applying visual effects to static gratings and are shown at their maximal distortion. **(A–C)** Effect of psilocin on visual discrimination in rats. **(A)** Timeline of the experiment, depicting training and testing phases. The correct ratio (bars, mean ± SEM) and decision time (lines, mean ± SEM) are shown for both motion-based (black) and luminance-based (gray) training trials over 5 days preceding testing, testing, and 1 day posttreatment. Testing sessions are color coded: red for motion-based and blue for luminance-based tasks. Both training and testing bars for the motion-based task are hatched to differentiate them from the luminance-based task. The first testing session, in which all responses were rewarded, is labeled as baseline. The number of trials per session is indicated in circles beneath the graph. Psilocin was administered on the final testing day, and its effects on task performance were evaluated. **(B)** Discrimination impairment and **(C)** time difference (decision time of saline minus psilocin) for luminance-based and motion-based tasks (luminance-based task, black bars; motion-based task, hatched bars). Data are presented as mean ± SEM. **(D)** Heat map showing the time spent in different maze regions averaged across all trials from the baseline session. The decision area is marked in yellow underneath. Statistical significance: ∗∗∗*p* < .001.

Rats were trained in a 2-choice visual discrimination task using a PVC-manufactured maze (Figure 2). The task progressed from a luminance-based task (black vs. white screens) to a motion-based task, where static cues consisted of vertical square-wave gratings, and dynamic cues were generated by applying 6 different visual effects to the static cue, distorting it to resemble hallucinatory motion. These effects maintained constant luminance and evolved smoothly over time (see the Supplement for details). Training followed a gradual increase in spatial frequency (0.095→0.135→0.175 cycles per degree). Each trial began when the maze door opened, which allowed the rat to enter and choose between 2 screens. A correct response triggered a cornball reward, while an incorrect response resulted in a 30-second confinement penalty. Rats were trained 20 trials per session, 5 sessions a week, until they achieved an 85% accuracy threshold over 3 consecutive sessions.

#### Testing Protocol

The 3-day testing phase assessed psilocin’s effect on visual discrimination. On the first day (baseline) (Figure 2A), rats completed 20 training trials followed by 24 testing trials, where incorrect responses were rewarded. On the second day, rats received saline and were tested under identical conditions, again with incorrect responses being rewarded. On the third day, psilocin (0.3 mg/kg) was administered, and testing occurred 30 minutes postinjection, with incorrect responses once again being rewarded to maintain motivation and engagement. This approach was essential to ensure that impairments reflected perceptual distortions rather than confusion from conflicting feedback. Because psilocin was expected to impair discrimination without altering the rats’ intent to perform the task correctly, rewarding incorrect responses prevented negative reinforcement, which could discourage participation or lead to task-irrelevant behaviors, complicating interpretation. A posttreatment training session was conducted the following day to verify that psilocin exposure did not impair task retention. After a 1-week washout period, rats were retrained on the luminance task and retested following the same protocol (see the Supplement for details).

### Data Analysis

In both humans and rats, the correct ratio was calculated as the proportion of correctly solved trials per session. Discrimination impairment was quantified as the absolute difference in correct ratios between the placebo/saline and psilocybin/psilocin conditions, as shown in the graphs. In rats, decision time—the time spent in a predefined decision area per trial (Figure 2D)—was extracted from above-view video recordings using EthoVision Pro v.3.1.1. The time difference was calculated as saline minus psilocin decision time. All data underwent Shapiro-Wilk’s normality test, Levene’s test, and Mauchly’s test where applicable. Significant main effects and interactions were followed by post hoc tests. Nonparametric alternatives were applied if assumptions for parametric tests were unmet. The alpha level was set at *p* < .05 (2 tailed). Statistical analyses were performed in STATISTICA v.13.3 (StatSoft, Inc.) and SPSS 25 (IBM Corp.). Data were presented as mean ± SEM or median (Q1/Q3) where appropriate. Areas under the curve (AUCs) for self-reported hallucination intensity and serum levels were calculated using GraphPad Prism 8.0, which was also used for graph generation. For a full description of the statistical procedures, see the Supplement.

## Results

### Human Experiment

Data from 15 participants who completed all 3 measurements were included in the final analysis, while 6 participants were excluded due to an inability to perform the task during the drug’s peak effect. The correct ratio differed significantly between placebo and psilocybin sessions (Wilcoxon signed-rank test, *n* = 15, *W* = 0, *z* = 3.408, *p* < .001), with psilocybin-intoxicated participants showing reduced accuracy in distinguishing static from dynamic cues (placebo: 98.7 ± 2.0%; psilocybin: 76.0 ± 10.8%). Discrimination impairment varied based on cue type and time since ingestion (Friedman’s analysis of variance, χ^2^_2_ = 14.550, *p* < .001), with the strongest effect observed 65 minutes postingestion (Figure 1A). At this time point, participants misclassified static cues as dynamic (Wilcoxon signed-rank test, *n* = 15, *W* = 1, *z* = 3.350, *p* < .001), particularly for facial stimuli (Wilcoxon signed-rank test, *n* = 15, *W* = 0, *z* = 2.934, *p* < .010). At 65 minutes, discrimination impairment was correlated with OSE (*r* = 0.652, *p* < .050), VUS (*r* = 0.623, *p* < .050), and psilocin serum levels (*r*_13_ = 0.585, *p* < .05). A correlation was also found between the AUCs of fitted psilocin serum levels and self-reported hallucination intensity (*r*_13_ = 0.547, *p* < .050) (Figure 1A). Discrimination impairment across time points is shown in Figure 1A, with differences between static/dynamic cues and face/ellipsoid stimuli presented in Figure 1C. Psilocybin significantly increased scores across all ASC subscales compared with placebo (*p* < .001, Bonferroni correction applied) (Figure 1B). Specific values were as follows: OSE (placebo: 2.5 ± 0.4%; psilocybin: 60.6 ± 3.3%), AIA (placebo: 1.1 ± 0.3%; psilocybin: 30.3 ± 2.0%), VUS (placebo: 2.2 ± 0.4%; psilocybin: 64.7 ± 2.7%), and the G-ASC score (placebo: 2.0 ± 0.4%; psilocybin: 50.6 ± 3.0%).

### Rat Experiment

Six rats were tested in both tasks. The correct ratio was significantly lower in psilocin trials than in saline trials (dependent Student’s *t* test, *t*_11_ = 4.693, *p* < .001), indicating impaired discrimination performance under psilocin (saline: 93.3 ± 5.7%; psilocin: 72.7 ± 1.8%). Decision time was inversely correlated with the correct ratio (Pearson product-moment correlation, *r* = −0.440, *p* < .050), suggesting that faster decisions were associated with greater impairment (Figure 2C, shown as decision time difference: saline minus psilocin). Discrimination impairment varied by task complexity, being significantly greater in the motion-based task than the luminance-based task (dependent Student’s *t* test, *t*_5_ = 4.067, *p* < .001) (Figure 2B).

## Discussion

Psychedelics are known to induce the illusion of motion in static objects and textures. In this study, we examined whether this effect occurs in both humans and rats following psilocin administration. Our findings indicate that both species experience motion-like distortions to a significant degree.

In humans, discrimination between static and dynamic cues remained impaired throughout the session but gradually diminished over time. High misclassification rates for static stimuli suggest that volunteers frequently perceived them as moving. This impairment closely paralleled psilocin plasma levels and self-reported hallucination intensity, with a significant correlation being observed between drug concentration and hallucinatory effects. Similarly, Madsen *et al.* (72) reported correlations between plasma psilocin levels, 5-HT_2A_ receptor occupancy, and subjective psychedelic intensity. Discrimination impairment was also linked to OSE and VUS—scales that measure visual alterations and mystical experiences (25). Notably, human faces were more likely to induce apparent motion than simple ellipsoids, suggesting that the complexity of visual stimuli modulates the extent of perceived distortions.

A review by Studerus *et al.* (27) of 8 psilocybin studies found that VUS effects varied with dose, with the subdimensions Elementary Hallucinations & Illusions and Scenery Hallucinations showing the highest scores, which increased from approximately 25% at low doses (0.12 mg/kg) to around 55% at high doses (0.315 mg/kg). In our study, volunteers reported even higher scores, 70% for Scenery Hallucinations and 76% for Elementary Hallucinations & Illusions after receiving 0.26 mg/kg. This discrepancy may stem from differences in the experimental environment because our visually enriched setting, which featured oriental rugs and bedspreads, might have amplified hallucinatory effects. However, the ASC questionnaire is completed retrospectively at the end of the session, whereas our visual discrimination paradigm enabled real-time assessment of visual distortions during intoxication. A similar approach was previously used in an early study by Siegel’s lab, where pretrained participants reported experiencing pulsating, explosive, and rotational movement patterns alongside lattice, tunnel, and kaleidoscopic formations (22).

Rats were tested in 2 distinct tasks compared to humans. As hypothesized, psilocin significantly impaired their ability to discriminate static from dynamic stimuli in the motion-based task, likely due to the drug-induced illusion of motion. The absence of significant effects in the luminance-based task suggests that this impairment stemmed from visual distortions rather than reduced motivation or cognitive deficits. A negative correlation between decision time and the correct ratio indicates difficulties in making accurate choices, likely reflecting psilocin’s influence on visual pattern recognition. However, when we compared long-term performance and decision times across tasks (Figure 2A), we found that rats generally performed better and responded faster in the luminance-based task, suggesting that motion-based discrimination was inherently more challenging, possibly due to stimulus complexity, as previously reported (73).

Furthermore, we observed a trend toward greater impairment and shorter decision times in rats trained to discriminate dynamic cues compared with rats trained to select static cues in the motion-based task (detailed in the Supplement). This trend is consistent with psilocin’s expected effect of inducing apparent motion on contrast patterns. Rats that discriminated dynamic cues might have perceived both stimuli as moving and reached a decision more quickly, while those trained to select static cues might have hesitated, perceiving both stimuli as incorrect and requiring more time to resolve the ambiguity. Additionally, performance was particularly impaired for stimuli modified by the wavewarp and ripples effects, which distorted the vertical grating in a way that could resemble visual disturbances induced by psilocin. While we cannot directly compare the quality of these visual alterations in rodents and humans, the use of these effects was designed to reflect the types of visual distortions often reported by humans under the influence of a psychedelic, such as the undulation of contours often described in psychedelic experiences.

The tendency of rats to misclassify static and dynamic cues, similar to that of humans, suggests that they likely experience comparable visual distortions. The highest error rates for wavewarp, ripple, and fisheye effects further indicate that these distortions may qualitatively resemble human hallucinations, such as the perception of vertical gratings as wobbling and buckling, as described by Oster (23). Unlike the study by Schmack *et al.* (74), which inferred ketamine-induced auditory hallucinations from confident false alarms after stimulus presentation, in our research, subjects responded during the presentation of stimuli, pointing to actual perceptual distortions as the most likely explanation.

With increasing doses, human participants report reduced motivation for movement and spatial coordination, accompanied by more intricate and immersive hallucinations (28). However, our study targeted hallucinations induced by moderate doses, ensuring that locomotion and spatial navigation remained intact for task performance. To achieve this, we conducted dose optimization prior to testing. While previous rat studies have administered psilocin at doses ranging from 0.2 to 4 mg/kg (63,75, 76, 77, 78), Winter *et al.* (78) found that a psilocybin dose of 0.8 mg/kg (equivalent to 0.57 mg/kg of psilocin) prevented rats from completing a 2-lever appetitive conditioning test. Similarly, our laboratory showed that 1 and 4 mg/kg of psilocin impair locomotion, whereas 0.25 mg/kg does not (63). Here, we demonstrated that 0.3 mg/kg of psilocin (equivalent to 0.42 mg/kg of psilocybin) effectively induced visual hallucinations in rats without compromising task performance. Other studies confirm that relatively low doses can impact cognitive function (76,78), challenging the recommended methodology for recalculating effective doses between humans and animals (79). This highlights the need for rigorous behavioral validation to establish appropriate cross-species dose equivalency (80).

A study by Carter *et al.* (81) demonstrated that psychedelics disrupted the balance of visual processing, selectively impairing global motion perception while preserving local motion processing. The rodent visual system, with its organization into 2 clusters that resemble the primate ventral and dorsal streams both anatomically (55,56) and functionally (57,58), provides a valuable model for studying visual hallucinations. Because rats can perform motion detection tasks (82,83), they could help confirm whether global motion perception is specifically impaired, as has been observed in humans. Furthermore, because the exact level of disruption within the visual pathway during hallucinations remains unclear, real-time tracking of stimulus propagation from the retina to higher cortical areas in rats could help pinpoint where visual distortions arise.

This study has several limitations. The visual tasks were designed to assess motion illusions in both species but did not account for other psychedelic-induced visual distortions, such as color or pattern changes. In rats, the small sample size and reliance on correct ratios and decision times as primary measures might have led us to fail to detect subtler perceptual changes. In humans, the exclusion of participants unable to complete the task at peak drug effects might have introduced selection bias, potentially underrepresenting those with more intense hallucinations. Addressing these limitations in future research could provide a more comprehensive understanding of drug-induced visual distortions.

### Conclusions

In this study, we directly assessed psilocin-induced visual-perceptual disturbances during intoxication in both humans and rats. Our results show that psilocin impaired the ability to distinguish between static and dynamic visual cues and, for the first time, provide evidence that rats experience visual distortions following psilocin administration. Although the quality of these visual distortions cannot be directly compared to those of humans, our findings suggest that both species experience alterations in visual perception while under the influence of psilocin, with similarities in the nature of these disturbances. The correlations between discrimination impairment, psilocin levels, and hallucination intensity in humans highlight the link between psilocin-induced changes and visual perception. Given the high prevalence of visual hallucinations in various neurological and psychiatric conditions (84, 85, 86, 87) and the limited understanding of their underlying mechanisms, establishing reliable preclinical models is essential (88). The rat model of hallucinations offers a valuable platform for investigating these phenomena and could contribute to the development and evaluation of novel treatments for disorders such as schizophrenia and Parkinson’s disease.

## Supplementary Material

Supplemental Methods and Materials, Results, and Figures S1–S2

Key Resources Table
