# Supplementary material for: Cross-Species Evidence for Psilocin-Induced Visual Distortions: Apparent Motion Is Perceived by Both Humans and Rats
Source: Biol Psychiatry Glob Open Sci. 2025 May 2;5(5):100524. doi: 10.1016/j.bpsgos.2025.100524 (PMC12209919; doi:10.1016/j.bpsgos.2025.100524)
Supplement: Supplemental Methods and Materials, Results, and Figures S1–S2 [file mmc1.pdf]

SUPPLEMENTARY INFORMATION

Cross-Species Evidence for Psilocin-Induced Visual Distortions: Apparent Motion Is Perceived by Both Humans and Rats

Vejmola *et al.*

Supplement

Methods and Materials

Human experiment

Study design and participants

This study was part of a double-blind, placebo-controlled crossover clinical trial (EudraCT No. 2012-004579-37), approved by the Ethical Committee of the National Institute of Mental Health (NIMH-CZ) and the State Institute for Drug Control. Twenty-one healthy volunteers (10 women), aged 28–53 years (mean = 37 ± 6.1), provided written informed consent. Psilocybin was administered orally at 0.26 mg/kg (equivalent to a full-hallucinogenic high dose (1,2)) using 1 mg and 5 mg capsules prepared at the Institute of Clinical and Experimental Medicine, Prague (IKEM). Identical placebo capsules contained *tritici amylum*. The minimum washout period between sessions was 28 days (mean = 49 days). For more details on study design, see (3).

Dosing

The formulations - capsules containing either 5 or 1 mg of psilocybin - were prepared in a pharmacy at IKEM (Institute of Clinical and Experimental Medicine in Prague). The dose of psilocybin was adjusted by combining capsules containing 1 and 5 mg, increasing or decreasing by 1 mg per 5 kg of body weight, with a 75 kg person being treated with 20 mg of psilocybin. The number of capsules was identical in the case of placebo (*tritici amylum*). The minimum time window between sessions per protocol was 28 days (mean = 49 days apart).

Visual task and stimuli

Volunteers performed the visual task at three different times: 65, 165, and 265 minutes after psilocybin administration; the task took six minutes. Either static or dynamic visual scenes lasting 8 seconds were presented. Dynamic cues were generated using Resolume Arena 6 software, applying eight visual effects („swirl,“ „bulge,“ „invert,“ „fractal 1/2,“ „waves,“ „graining,“ and „breakdown“) that distorted the static cue – images of different people's faces (man/woman) or white ellipsoid in a black background (see Figure 1, upper part). The stimuli were presented randomly so that each scene appeared eight times

(8x static ellipsoid, 8x dynamic ellipsoid, 8x static face, 8x dynamic face). After the presentation of each scene, the volunteer had to select with the mouse whether the object was changing or not. The specific assignment was as follows: „In a moment, you will be presented with dynamic and static images. Your task is to decide whether the image moves or not." After each stimulus presentation, the subject was asked to respond: the object was moving (left button); the object was not moving (right button). Stimuli were presented via OpenSesame software on a 22-inch Full HD LCD screen from a distance of 70 cm at a viewing angle of 90°.

Acute subjective drug effects

Subjective acute drug effects were assessed using the ASC scale (4), which subjects were asked to complete immediately after the psilocybin session (380 mins after ingestion). The ASC contains 72 dichotomous items covering a broad range of phenomena potentially present during altered states of consciousness. The subjects responded to each question by placing a mark on a horizontal visual analog scale. Four scales were derived: Oceanic Boundlessness (OSE), Dread of Ego Dissolution (AIA), and Visionary Restructuralization (VUS). G-ASC scale represents the overall (General) magnitude of the effect comprising all of the questionnaire items.

At the same time, volunteers were asked to plot their visual experience as a curve on a continuous visual analog scale (x-axis time, 0-7 hours; y-axis intensity, 0-10) according to the following assignment: „Create a continuous graph of how much illusion or hallucination was present. Up to intensity 4, these are only pseudo-hallucinations (or synesthesia, etc.); from intensity 4 onwards, there are true hallucinations (you see things that others do not see, etc.). Intensity 10 means that you are completely disoriented in space-time." The plotted curves were then digitized with the freely available Webplotdigitizer tool (5,6) using the „X step w/ Interpolation" algorithm with the 0.1 step and 200 % of ΔX smoothing. See Figure S1 for all the individual graphs.

Determination of psilocin in human serum by LC-MS/MS

Sample preparation

After ingestion, psilocybin is rapidly dephosphorylated and becomes psychoactive psilocin (9). Blood samples were taken from a cannula and inserted over the session at 1, 2, 4, 6, and 24 hours after ingestion of the capsules. Blood was immediately centrifuged for 10 minutes at 10°C at 1520 RCF; serum was then stored at -80°C until analyzed. The samples were prepared using two different procedures to determine free and total psilocin in human serum. Each sample was prepared in duplicate.

For determination of free psilocin, 200 µL of serum was diluted with 200 µL of 0.1% (v/v) acetic acid in the water, mixed and 5 µL of internal deuterated standard (2 µg/mL of psilocin-d10 in acetonitrile) was added and mixed again. Subsequently, the solution was precipitated by two sequential additions of 400 µL of acetonitrile. After centrifugation, an aliquot of 960 µL was taken and evaporated using a vacuum concentrator. Finally, 250 µL of 5% (v/v) methanol with the addition of 10 mmol/L formic acid in water was used for reconstitution, mixed, centrifuged, and analyzed using LC-MS/MS.

The procedure with hydrolysis for determination of total psilocin in serum utilized beta-glucuronidase: 10 µL of 0.1 mmol/l ascorbic acid and 5 µL of internal standard (2 µg/mL of psilocin-d10 in acetonitrile) were added to 100 µL of serum and mixed. Subsequently, 290 µL of 50 mmol/L acetic buffer (pH 4.5) with 500 U of beta-glucuronidase was added, mixed, and the sample was incubated at 37 °C for 4 hours. Then, the procedure followed the steps described to determine free psilocin, i.e., sequential addition of acetonitrile, centrifugation, evaporation, and reconstitution.

Matrix calibration was prepared and used for quantification, i.e., blank serum was enriched by the standard of psilocin to obtain the required concentration levels. The procedure treated calibration samples without hydrolysis.

UHPLC-MS/MS conditions

A 1290 Infinity liquid chromatography (LC) system was utilized with a guard column equipped with a Zorbax Eclipse Plus C18 column (50 mm × 2.1 mm, 1.8 µm particle size). Mobile phases were 10 mmol/L formic acid in water (mobile phase A) and MeOH (mobile phase B). The flow rate was 0.25

mL/min, the column temperature was maintained at 35 °C, and the autosampler was set at 5 °C. The time profile of the gradient was as follows: mobile phase composition started at 5% B (v/v), then the amount of B was increased in two linear steps, firstly to 45% B at 120 seconds, secondly to 100% B at 180 seconds, this composition was kept constant for 78 seconds, and then the column was re-equilibrated for 150 seconds to starting conditions, the total run time was 450 seconds. A 6460 QqQ mass spectrometer (Agilent Technologies, USA) in positive electrospray (ESI +) ionization mode was used for detection. Ion source settings were as follows: gas temperature 320 °C, gas flow 12 L/min, nebulizer 50 psi, sheath gas temperature 400 °C, sheath gas flow 10 L/min, capillary voltage 3800 V.

Distinct data points were then curve-fitted in a Matlab Curve Fitting Toolbox with a 1-compartment model with a first-order absorption equation for a single dose oral administration ((F*D*ka)/V*(ka-ke))*(exp(-ke*x)-exp(-ka*x)) (10). See Figure S1 for all individual values and fitted curves.

Rat experiment

Animals

Due to the strong influence of the estrous cycle phase on females' responses to the effects of psychedelics (7,8), only males were included in this experiment. Male Long Evans rats (N = 10) were used, kept in pairs in standard plastic breeding containers (enriched with chew toys and PVC tubes) in an air-conditioned room with a controlled temperature (± 22 °C) and humidity (± 40 %) with a regular twelve-hour (6:00 a.m.–6:00 p.m.) light/dark cycle. Food intake was restricted to keep rats motivated to respond to food rewards. The food was removed in the morning (± 7:00 a.m.) and returned in the afternoon after training. Animal weights were monitored throughout the experiment to ensure they did not drop below 90 % of their unrestricted feeding weight. Access to water was *ad libitum*. The principles of the National Committee for the Care and Use of Laboratory Animals, CZ, and European Union guidelines (86/609/EU) were adhered to. The experiment was approved by the Czech National Committee for the Care and Use of Laboratory Animals (reference: MEYSCR36607/2018–4).

Drugs

Psilocin was synthesized and supplied by the Forensic Laboratory of Biologically Active Compounds, Department of Chemistry of Natural Compounds, University of Chemistry and Technology, Prague, Czechia. Psilocin was dissolved in saline (0.9% NaCl) and acidified with a tiny amount of glacial acetic acid (5 µL per 20 mL). Psilocin 0.3 mg/kg or saline was injected subcutaneously in a 2 mL/kg volume. The solution was prepared fresh on the day of testing.

Visual task and stimuli

In rats, two types of visual tasks were used. The luminance-based task involved black or white cues, while the motion-based task featured either static or dynamic cues. The static cue was a square-wave vertical black-and-white grating with full contrast and a spatial frequency of 0.175 cycles per degree (c/d). Dynamic cues were generated using Resolume Arena 6 software, applying six visual effects („twisted," „wavewarp," „ripples," „fisheye," „blur," and „noisy") that distorted the static cue to simulate the range of hallucinatory movements reported by intoxicated people. These effects maintained constant screen luminance and ran smoothly in a loop, gradually developing and fading. The entire experimental setup was programmed in Python, with stimuli presented on two 10.1" Full HD LCD monitors viewed from a distance of 500 mm. Details are shown in the upper part of Figure 2.

Training procedure

A detailed representation and description of the PVC-manufactured maze is shown in the upper part of Figure 2. The maze was placed in a sound-attenuated room (with a 40 dB white noise background) with uniform lighting (5 lx at the maze's bottom). A single person operated the entire experiment, from acclimatization to testing. Ten handled rats, starting at 5 weeks old, began the experiment with a week of acclimatization, during which they were allowed to freely explore the apparatus and collect cornballs placed near the reward tube. Once all the cornballs were collected, the rats were removed.

Initially, the rats underwent a shaping procedure to discriminate between full-black and full-white screens (luminance-based task), learning that a specific image on one screen predicted the delivery of a cornball in the adjacent tube. The presentation of stimuli was randomized in blocks of six. Each trial

began with the entrance door opening, allowing the rat to enter the maze, after which the door was closed. The rat then navigated to the arm crossing, choosing either the left or right arm. A correct choice was rewarded with a cornball, while an incorrect response resulted in a 30-second penalty of being closed in the arm without a reward. A decision was considered definite when the rat fully crossed the entrance edge of the chosen arm. After eating the cornball or serving the penalty, the rat was removed and immediately presented with the next trial. The rats were divided into two groups, with 5 discriminating black screens and 5 white. Each training session consisted of 20 trials, conducted in the afternoon (2:00 p.m.–6:00 p.m.). The apparatus was disinfected with a 70% EtOH solution after each rat. Training occurred once daily, 5 days a week (Monday–Friday). Within four weeks (20 training sessions), all ten rats achieved a stable success rate of at least 85% over three consecutive sessions.

After mastering the luminance-based task, rats were advanced to a motion-based task, with dynamic/static cues starting at a spatial frequency of 0.095 c/d. Within 50 training sessions, six rats successfully mastered the pattern-based task, maintaining at least 85% success over three consecutive sessions. These six rats (3 discriminating static, 3 dynamic scenes) continued training, with the spatial frequency increased to 0.135 c/d in the first week and then to 0.175 c/d the following week.

Dose optimization

To test psilocin in our behavioral paradigm, we conducted dose optimization on four rats that had not mastered the motion-based task and were not included in the final testing. We tested doses of 1 mg/kg, 0.5 mg/kg, 0.4 mg/kg, and 0.3 mg/kg on their performance in a luminance-based task. The 0.3 mg/kg dose was identified as the highest at which the rats could fully complete the task. At 0.4 mg/kg, they either completed the task partially or with significant delays, and at 0.5 mg/kg and above, the rats were unable to solve the task at all.

Testing

After stabilization of the behavioral response (success rate of at least 85 %), six rats underwent the final phase of the experiment. Rats were tested twice per day in a row on three consecutive days: the first training session, with 20 trials, and the second testing session, with 24 trials. Within 24-trial sessions,

rats were rewarded for incorrect responses on the first day. Since this experiment assumed that the rats would make incorrect decisions while believing they were solving the task correctly, they should not be penalized for this response. Introducing the reward for incorrect decisions was crucial to maintaining the rats' motivation and ensuring that the testing results reflect the effects of psilocin rather than the impact of penalties. Performance in this control session thus indicates whether rats can solve the same task twice in a row and whether rewarding incorrect responses impacts overall performance. On the second day, rats were administered a vehicle and rewarded also for incorrect responses. Finally, on the third day, rats were administered psilocin, tested 30 minutes after application, and rewarded for incorrect responses again. A single training session took place the day post-treatment to test if psilocin exposition has a retrieval effect on task performance. The long-term performance of the rats during the training sessions, five days before and the day after testing with the timeline of the experiment is shown in Figure 2. After a one-week washout period, rats were retrained to the initial luminance-based task. After stabilization of the behavioral response, they underwent the same testing protocol.

Data analysis

In both humans and rats, the *correct ratio* was calculated for each session as the proportion of correctly solved trials divided by the overall number of trials. *Discrimination impairment,* expressed as the absolute difference in correct ratios across the placebo/saline and psilocybin/psilocin sessions (placebo/saline minus psilocybin/psilocin), was calculated and presented in the graphs. In rats, we also evaluated *decision time* extracted from the above-view video records using the EthoVision Pro v. 3.1.1 software (Noldus, Netherlands). It was defined as the average time spent in a predefined decision area (Figure 2F) per trial across the session. The time difference was calculated as the decision time of saline minus psilocin. All data were subjected to Shapiro-Wilk's Normality test, Levene's test, and, where appropriate, Mauchly's Test. Significant main effects and interactions were followed by an appropriate *post hoc* test where it is relevant. Nonparametric analogs were used when the assumptions for parametric tests were not met. Alpha level was set up at $p < 0.05$, two-tailed. Analyses were conducted using the software STATISTICA version 13.3. [StatSoft, Inc. (2010)] and IBM SPSS 25. Data were presented as mean ± the standard error mean (SEM) or median with the first/third quartile (Q1/Q3), respectively. The

areas under the curves (AUC) for self-reported hallucination intensity and serum levels were calculated using GraphPad Prism 8.0, which was also employed to generate all the graphs.

Human experiment

The effect of psilocybin on general cue discrimination was evaluated using the Wilcoxon signed-rank test, with treatment (psilocybin *versus* placebo) as the independent variable and *correct ratio* as the dependent variable. Additionally, Friedman's ANOVA was employed to assess the impact of cue type (face *versus* oval), the time elapsed since psilocybin administration (T65, T165, T265), and their interaction on *discrimination impairment*. Spearman rank correlation was used to examine the relationships between *discrimination impairment*, ASC scale scores, and psilocybin serum levels, the same as the relationship between the areas under the curve for serum levels and self-reported hallucination intensity. Finally, paired t-tests with Bonferroni correction for multiple comparisons were applied to compare the ASC scale values (OSE, AIA, VUS, and VWB) between psilocybin and placebo sessions.

Rat experiment

Dependent Student's t-tests were used to assess the effect of psilocybin on *correct ratio* and evaluate the effect of cue complexity (luminance- *versus* motion-based cues) on *discrimination impairment.* The effects of the dynamic character of motion-based cues (static *versus* dynamic) and the various cue subtypes (black *versus* white or twisted *versus* wavewarp/ripples/fisheye/blur/noisy) on *discrimination impairment* were analyzed using Independent Student's t-tests. Pearson product-moment correlation was employed to examine the relationship between *decision time* and *correct ratio.*

Results

Rat experiment

No significant differences were observed for luminance-based cues (black vs. white; Independent Student's t-test, t(4) = -0.230, p = 0.830) or among motion-based cue subtypes (twisted, wavewarp, ripples, fisheye, blur, noisy; ANOVA, F(5,30) = 1.064, p > 0.050). Within the motion-based task, rats trained to respond to dynamic cues showed a trend toward greater impairment than those trained to static cues (Independent Student's t-test, t(4) = 2.683, p = 0.055, Figure S2).

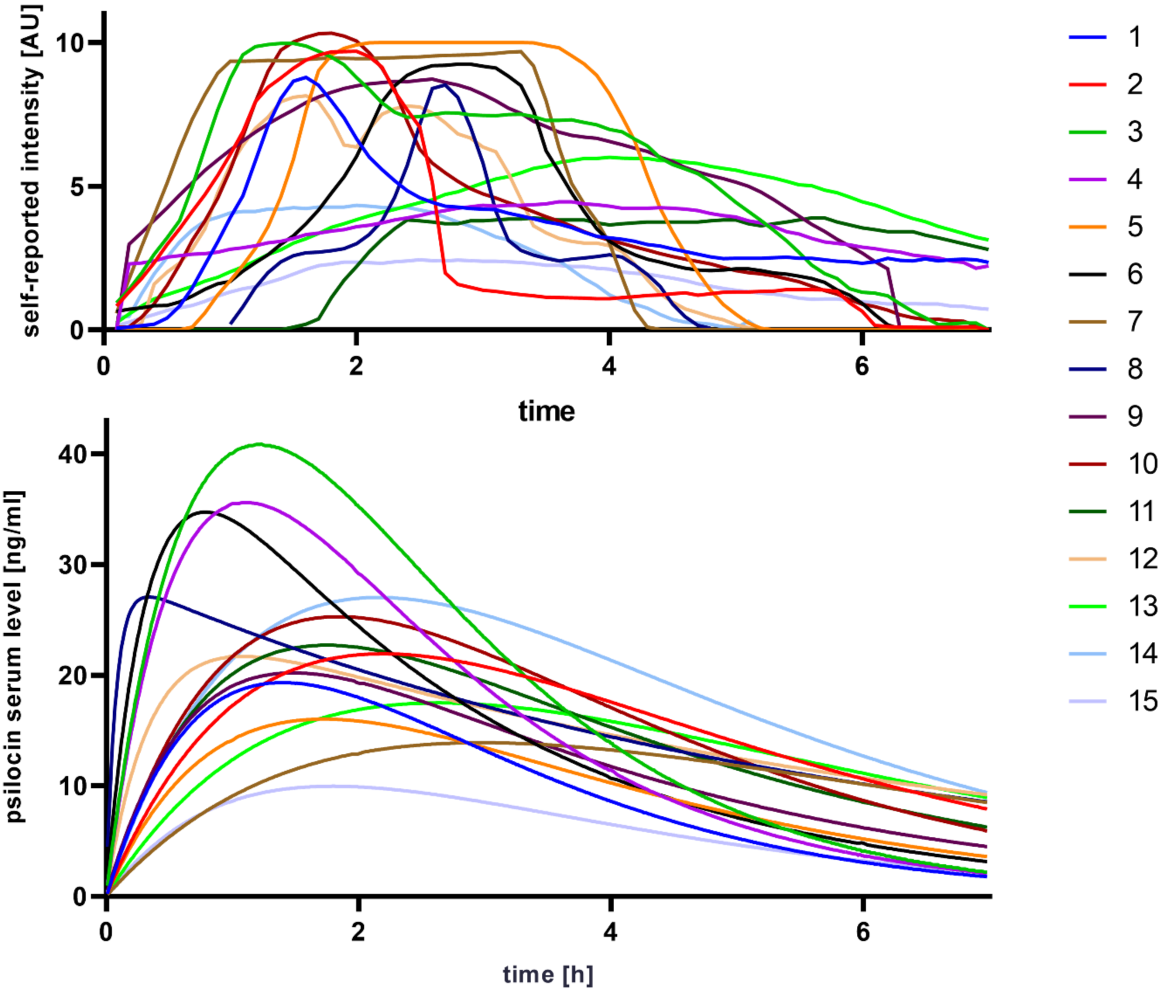

Figure S1: Individual values of all volunteers of self-reported intensity of hallucinations (**upper**) and fitted curves of psilocin serum levels (**lower**).

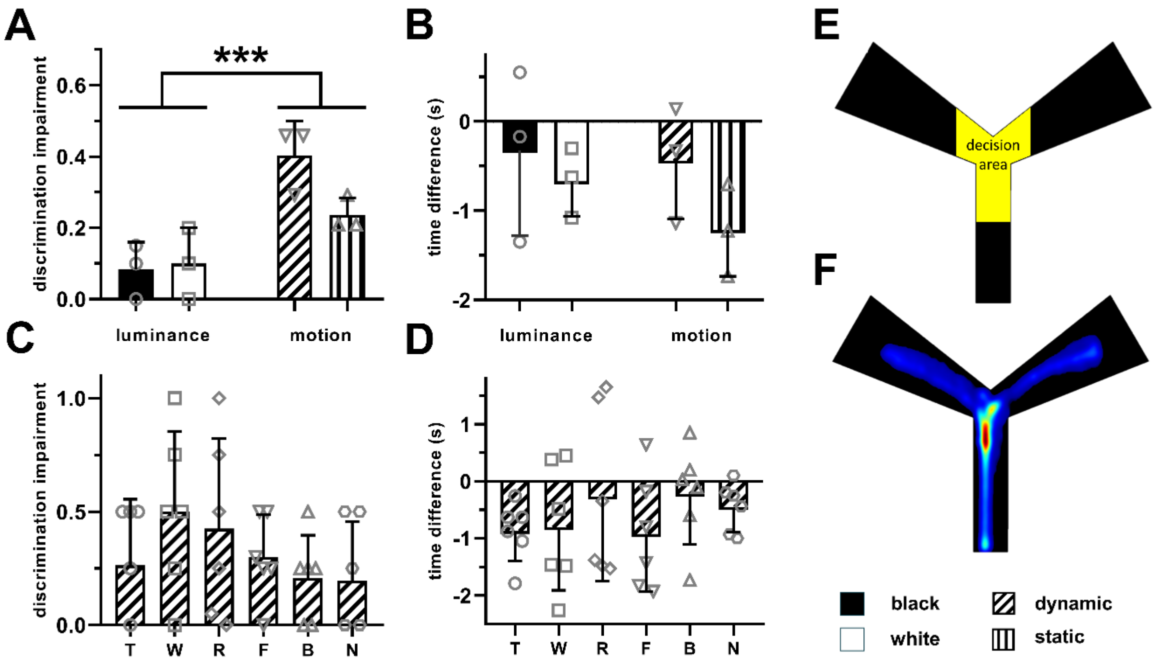

Figure S2: Rat Experiment. (**A, B**) Discrimination impairment (**A**) and time difference (**B**; decision time of saline minus psilocin) for luminance-based (black and white) and motion-based tasks (static cues: vertical hatching; dynamic cues: angled hatching). Data are presented as mean ± SEM. (**C, D**) Discrimination impairment (**C**) and time difference (**D**) for specific types of dynamic visual cues: T = twisted, W = wavewarp, R = ripples, F = fisheye, B = blur, N = noisy. (**E, F**) (**E**) Maze floorplan with the decision area highlighted in yellow. (**F**) Heat map showing the time spent in different maze regions, averaged across all trials from the baseline session. Statistical significance: $*p < 0.05$; $**p < 0.01$; $***p < 0.001$.

References

1. Studerus E, Kometer M, Hasler F, Vollenweider FX (2011): Acute, subacute and long-term subjective effects of psilocybin in healthy humans: a pooled analysis of experimental studies. *J Psychopharmacol* 25: 1434–1452.

2. Vollenweider FX, Kometer M (2010): The neurobiology of psychedelic drugs: implications for the treatment of mood disorders. *Nature* 11: 642–651.

3. Bravermanová A, Viktorinová M, Tylš F, Novák T, Androvičová R, Korčák J, *et al.* (2018):

Psilocybin disrupts sensory and higher order cognitive processing but not pre-attentive cognitive processing—study on P300 and mismatch negativity in healthy volunteers. *Psychopharmacology (Berl)* 235: 491–503.

4. Dittrich A (1998): The standardized psychometric assessment of altered states of consciousness (ASCs) in humans. *Pharmacopsychiatry* 31: 80–84.

5. Marin F, Rohatgi A, Charlot S (2017): WebPlotDigitizer, a polyvalent and free software to extract spectra from old astronomical publications: application to ultraviolet spectropolarimetry. Retrieved from http://arxiv.org/abs/1708.02025

6. Rohatgi A (2022): Webplotdigitizer [no. 4.6]. Pacifica, California, USA. Retrieved from https://automeris.io/WebPlotDigitizer

7. Páleníček T, Hliňák Z, Bubeníková-Valešová V, Novák T, Horáček J (2010): Sex differences in the effects of N,N-diethyllysergamide (LSD) on behavioural activity and prepulse inhibition. *Prog Neuro-Psychopharmacology Biol Psychiatry* 34: 588–596.

8. Tylš F, Páleníček T, Kadeřábek L, Lipski M, Kubešová A, Horáček J (2015): Sex differences and serotonergic mechanisms in the behavioural effects of psilocin. *Behav Pharmacol* 1.

9. Hasler F, Bourquin D, Brenneisen R, Bär T, Vollenweider FX (1997): Determination of psilocin and 4-hydroxyindole-3-acetic acid in plasma by HPLC-ECD and pharmacokinetic profiles of oral and intravenous psilocybin in man. *Pharm Acta Helv* 72: 175–84.

10. Wijnand HP (1988): Pharmacokinetic model equations for the one- and two-compartment models with first-order processes in which the absorption and exponential elimination or distribution rate constants are equal. *J Pharmacokinet Biopharm* 16: 109–128.
